# Altered Macular Vasculature in Migraine Patients without Aura: Is It Associated with Ocular Vasculature and White Matter Hyperintensities?

**DOI:** 10.1155/2020/3412490

**Published:** 2020-04-13

**Authors:** Nurdan Gamze Taşlı, Alevtina Ersoy

**Affiliations:** ^1^Department of Ophthalmology, Erzincan Binali Yıldırım University Hospital, College of Medicine, Erzincan, Turkey; ^2^Department of Neurology, Erzincan Binali Yıldırım University Hospital, College of Medicine, Erzincan, Turkey

## Abstract

**Aim:**

We aimed to determine the alterations in macular and optic nerve vasculature in patients with migraine without aura using optical coherence tomography-angiography (OCTA). We also aimed to determine whether there were clinical differences and alterations in ocular structures in migraine cases with white matter hyperintensities (WMH) using magnetic resonance imaging (MRI). *Materials and Methods*. The study group comprised patients with migraine without aura and age- and sex-matched healthy controls. Detailed histories of the patients with migraine were recorded including the disease duration, number of attacks in the last month, and attack durations. Visual evoked potentials (VEP) were recorded in all migraine patients. The migraine disability assessment (MIDAS) questionnaire was administered to all patients. The patients were divided into two groups as migraine with WMHs and migraine without WMHs. All subjects underwent a complete neurological and ophthalmological examination. Only the right eyes of the patients were included in the study. Retinal imaging was performed using OCT and OCTA.

**Results:**

A total of 66 migraine patients (29 with WMH and 37 without WMH) and 43 healthy controls were included in this study. Among the migraine patients, disease duration, attack frequency in the last month, attack durations, and the visual analogue scale (VAS), MIDAS, and VEP scores were all similar between those with and without WMHs. There was no significant difference between the groups regarding the ganglion cell complex, foveal, and retinal nerve fiber layer thicknesses. The superficial or deep vascular perfusion densities of the optic disc were also similar between the groups. The foveal avascular zone (FAZ) was significantly larger (*P*=0.034), and both superficial and deep macular vascular densities were significantly lower in the migraine groups compared with the healthy controls (*P*=0.001). There was no significant difference concerning the FAZ size or vascular densities between the migraine groups with and without WMHs. In the correlation analysis performed between the migraine patients, the FAZ size was correlated with age and VAS and MIDAS scores while both superficial and deep macular vascular densities were negatively correlated with age and VAS and MIDAS scores.

**Conclusion:**

We suggest that for not only migraine with aura but also migraine without aura, neurovascular structures play an important role in pathogenesis, and novel studies are warranted to elucidate the alterations in these and determine the significance of WMHs in these patient groups.

## 1. Introduction

Migraine is one of the most common neurological diseases with lifetime prevalence reaching 33% in females and 13% in males [1]. In one-third of migraine patients, headaches are preceded by focal neurological disorders, usually visual, which is known as “aura” [2]. Migraine with aura is reported to be associated with massive changes in cortical and retinal perfusion [3, 4].

Brain white matter hyperintensities (WMHs) visualized using magnetic resonance imaging (MRI) have been described in migraine, as well as in several other neurological disorders [5]. Although the cause and mechanisms of these lesions are still open to debate, they have been considered as an indirect marker of focal cerebral hypoperfusion induced by migraine attacks, particularly if repeated and associated with an aura [6].

Migraine has been suggested as a risk factor for ischemic complications of the retina and optic nerve for years [7, 8]. Ophthalmic disorders associated with migraine include branch and central retinal artery and vein occlusions, as well as anterior and posterior ischemic optic neuropathy [9, 10]. Besides, migraine has been reported to be a risk factor for the diagnosis and progression of normal tension glaucoma [11].

Optical coherence tomography-angiography (OCTA) is a method of generating three-dimensional images of vasculature in vivo without dye injection [12]. An advantage of OCTA is that it allows discrimination and evaluation of the superficial and deep capillary plexus networks [13].

In this study, we aimed to determine the macular and optic nerve vasculature in patients with migraine without aura using OCTA and compare the results with healthy controls. Also, we evaluated the structural parameters of the macula and optic nerve using spectral-domain optical coherence tomography (OCT). Due to the potential risk of systemic and ocular ischemic events in migraine patients, we hypothesized that the eyes of these patients would have an increased foveal avascular zone (FAZ) area when compared with the controls. Besides, we hypothesized that the migraine patients would have decreased vessel density (VD) in the macula and optic nerve, as well as decreased foveal, ganglion cell complex (GCC), and retinal nerve fiber layer (RNFL) thicknesses. We aimed to reveal the vascular alterations in migraine patients without aura and compare our results with similar studies in the literature. We also aimed to determine whether there were clinical differences and alterations in the ocular structures of migraine patients with and without WMHs using MRI.

## 2. Materials and Methods

This cross-sectional study adhered to the tenets of the Declaration of Helsinki, and informed consent was obtained from all patients. The study was approved by the local ethics committee (E. 13164), and informed consent was obtained from all participants. The study group comprised migraine patients without aura and age- and sex-matched healthy controls. The patients that had been diagnosed according to the revised criteria of the International Classification of Headache Disorders, Second Edition (ICDH-II), were recruited from our neurology clinic [14]. Detailed histories of the patients with migraine were recorded including the disease duration, number of attacks in the last month, and attack durations. Visual evoked potential (VEP) latencies were recorded in all migraine patients. Also, the migraine disability assessment (MIDAS) questionnaire was administered to all patients [15]. The patients were divided into two groups as migraine with WMHs and migraine without WMHs. Recently performed (within the last two months) brain MRI findings of all migraine patients were examined, and the presence of WMHs was recorded. The Fazekas scale was used to assess the severity of WMHs. Periventricular and deep WMHs were evaluated separately and summed to obtain the Fazekas scores, which were then used to determine the degree of WMH severity (mild: 0–2; moderate: 3-4; severe: 5-6) [16]. All subjects were evaluated by the same neurologist and ophthalmologist and underwent a complete neurological and ophthalmological examination. Only the right eyes of all patients were included in the study.

The exclusion criteria for all groups included any neurologic disorder other than migraine (including neurodegenerative diseases, such as Alzheimer's or Parkinson's diseases), any disorder of the optic nerve (including glaucoma) or retina, history of intraocular surgery other than cataract extraction, a refractive error greater than 3.00 or less than 6.00 dioptres, systemic conditions affecting the microvasculature, such as diabetes mellitus, hypertension, vasculitis, or renal disease, and ocular media opacity precluding high-quality imaging. Healthy controls were also excluded if they were taking vasoactive medications, such as calcium channel blockers.

During the ophthalmological examination, best-corrected visual acuity (BCVA) testing using the Snellen chart, slit-lamp biomicroscopy, intraocular pressure (IOP) measurement, dilated fundus examination (using a 90-dioptre lens), and intraocular pressure (IOP) measurement were performed in all participants. Retinal imaging was undertaken using OCTA (RS-3000 Advance, Nidek Co., Tokyo, Japan) and OCT (Nidek RS-3000 Advance (Nidek Co., Tokyo, Japan).

### 2.1. OCT

The measurements of the macular and RNFL thicknesses were obtained by a Nidek RS-3000 Advance (Nidek Co., Tokyo, Japan) device, operating at a central wavelength of 880 nm and a speed of an A-scan rate of 53,000 seconds/s. The axial and transverse scan resolution in tissue was 7 and 20 *μ*m, respectively. The RNFL thickness was recorded at four different quadrants using the TSNIT chart. The macular map (ETDRS chart) was used to obtain the macular thickness. The GCC thickness was recorded for the whole macula, as well as the superior/inferior macular sectors.

### 2.2. OCTA

All OCTA images were acquired using the same device. The images included a 3 × 3 mm^2^ area centered on the fovea and a 2.4 × 4 mm^2^ measurement area centered on the optic nerve head. NIDEK recently developed a new version of OCTA analyzing software (ver. 1.1.5) that sets the margins of FAZ and calculates the FAZ area automatically. This software also allows measuring the macular vascular/perfusion densities for nine sectors following ETDRS. With this new update, the peripapillary vascular/perfusion densities are automatically divided into superior/inferior (S/I) sectors, as well as eight sectors according to the TSNIT pattern. In this study, we used the perfusion density (PD) to analyze the retinal vascular network. The quantitative analysis of PD was performed using the PD maps of the macula (ETDRS chart) and the optic nerve head (S/I and TSNIT charts). With the macular OCTA scans, the FAZ area on the superficial capillary plexus (SCP) and the PD values of the whole SCP, the inner (0.5 to 1.5 mm) and outer (1.5 to 3.00 mm) ETDRS sectors of SCP, the nine ETDRS sectors, the whole deep capillary plexus (DCP), and the inner and outer ETDRS sectors of DCP were collected. The PDs of the retinal peripapillary capillary plexus (RPCP) for the S/I sectors and TSNIT sectors were also recorded.

**Figure 1:**
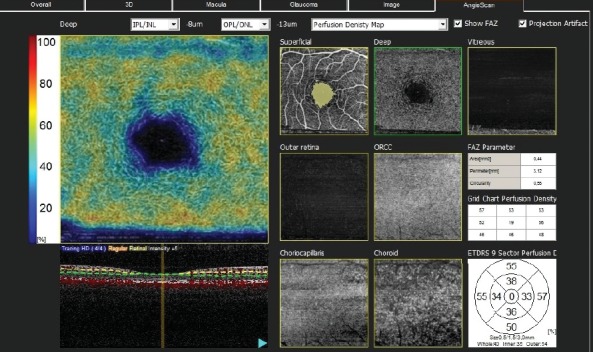
The quantitative analysis of vascular densities (VDs) performed using the colored VD maps of the macula (ETDRS chart) and macular OCT-A scans. The perfusion densities of the outer (1.5 to 3.00 mm) ETDRS sectors, nine ETDRS sectors, the whole deep capillary plexus (DCP), and the inner and outer ETDRS sectors of DCP were obtained. The foveal avascular zone on the superficial capillary plexus (SCP) and the PDs of the whole SCP and the inner SCP (0.5 to 1.5 mm retinal peripapillary capillary plexus were also recorded for the superior and inferior sectors and TSNIT sectors.

**Figure 2:**
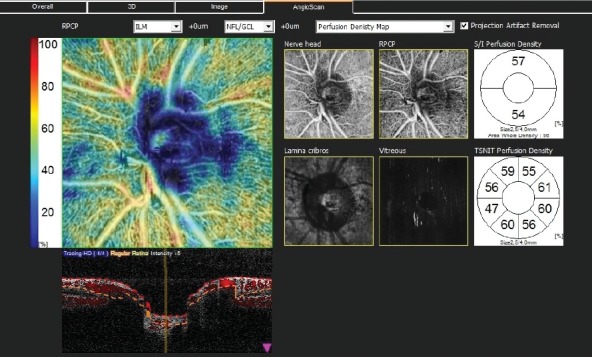
Peripapillary vascular/perfusion densities automatically divided into superior and inferior sectors and eight sectors according to the TSNIT pattern.

**Figure 3:**
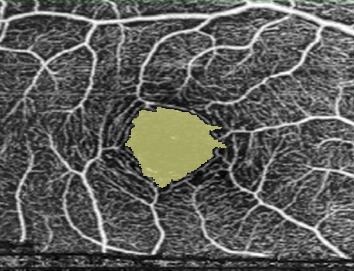
Macular OCT-A scans of the foveal avascular zone.

GCLT was recorded for the S/I sectors of the macula. Vascular density (VD) was defined as the percentage of vascularized tissue within the surrounding area. The quantitative analysis of VDs was performed using the colored VD maps of the macula (ETDRS chart) (Figure 1) and the optic nerve head (TSNIT chart) (Figure 2). Using the macular OCTA scans, the FAZ area (Figure 3), perimeter and circularity index (CI, values closer to “1” indicating a higher circularity) at the level of SCP, and the VDs of SCP and DCP were recorded. Automated segmentation defined the en face slab for the superficial retinal layer to extend from the internal limiting membrane to 13 m below the inner nuclear layer. The enface slab for the deep retinal layer extended from 8 m below the inner nuclear layer to 13 m below the outer nuclear layer. The software also provides the segmentation of the outer retina, choriocapillaris, and choroid.

### 2.3. Statistical Analyses

Statistical analyses were performed using SPSS software version 20.0 (SPSS, Inc., Chicago, IL, USA). Continuous variables were presented as mean ± standard deviation (SD). The one-way analysis of variance was used to compare the variables between the groups. The correlations of FAZ and the VDs of SCP and DCP with age, disease duration (years), attack frequency (last month), attack duration (hours), VAS, MIDAS scores, and VEP were determined. Clinical characteristics were analyzed using the Pearson correlation coefficients in the migraine groups. *P* < 0.05 was considered statistically significant. A power calculation was not required due to the exploratory nature of the study.

## 3. Results

A total of 66 migraine patients (29 with WMHs and 37 without WMHs) and 43 age- and gender-matched healthy controls were included in this study (Table 1). Among the migraine patients, disease duration, attack frequency in the last month, attack durations, and the VAS, MIDAS, and VEP scores were all similar between those with and without WMHs (Table 2).

There was not any significant difference between the groups regarding the GCC, foveal, and RNFL thicknesses (Table 3). Similarly, no significant difference was found between the groups regarding the superficial or deep PDs of the optic disc (Table 4).

The FAZ size was significantly larger (*P*=0.034), and both superficial and deep macular VDs were significantly lower in the migraine groups compared with the healthy controls (*P*=0.001). However, there was no significant difference concerning the FAZ size or VDs between the migraine groups with and without WMHs (Table 5).

The correlation analysis performed among the migraine patients revealed that the FAZ size was correlated with age and VAS and MIDAS scores while both superficial and deep macular VDs were negatively correlated with age and VAS and MIDAS scores (Table 6).

## 4. Discussion

Migraine is a common disease with many systemic vascular alterations, but these alterations were reported to be more commonly present in patients with aura. In this study, we analyzed the optic disc and macular VDs in migraine patients without aura, and we did not find any alteration in the VDs of the optic disc, but the FAZ size was significantly larger and both superficial and deep macular VDs were significantly lower in migraine patients without aura compared with the healthy controls. We also determined that the presence of WMHs did not result in any alteration regarding the optic disc or macular VDs in migraine patients without aura. In the correlation analysis, the FAZ size was significantly correlated with age and VAS and MIDAS scores, while the macular VDs were negatively correlated with these clinical parameters.

The pathophysiological mechanisms underlying migraine are still not clear; however, the alterations in the ocular posterior structure have been previously reported to indicate transneuronal retrograde degeneration of the primary visual cortex and cortical spreading depression in migraine patients. Gipponi et al. [17] reported decreased RNFL thickness in the superior retinal quadrant in migraine patients compared with the normal subjects, which did not depend on illness duration or frequency. Demirci et al. [18] reported significantly thinner RNFL values in migraine patients compared with the healthy controls. They also noted that this reduction was more prominent in migraine patients that smoked. Demircan et al. [19] showed that the mean RNFL thickness for the nasal and nasal inferior sectors, the mean choroid thickness, and the foveal thickness were significantly smaller in the migraine patients with and without aura compared with the controls; however, the mean macular thickness did not significantly differ between the groups. Acer et al. [20] reported that the RNFL thickness was significantly reduced in the temporal and nasal superior sectors in the migraine group without aura, but there was no significant difference regarding the GCC and macular thicknesses between the patients and controls. The authors also stated that the ocular pulse amplitude did not significantly differ between groups and concluded that although there was sectorial RNFL thinning in migraine patients without aura, the pulsative choroidal blood flow may not be affected during the chronic course of the disease. Reggio et al. [21] reported significant thinning in RNFL, GCC, and choroidal in migraine patients compared with the healthy controls. Ao et al. [22] found that the nasal peripapillary RNFL and inferior inner macular layer were significantly thinner in the migraine group with aura, but there was no difference between the two migraine groups and the control group. In contrast, there are also studies reporting no differences between the migraine patients and control cases regarding the RNFL thickness. For example, in a large study conducted with 3,224 eyes of 1,973 subjects [23], no significant correlation was observed between the presence of migraine and the RNFL thickness. Salman et al. [24] also reported that the RNFL thickness of the migraine group was not statistically significantly different from that of the control group. Similarly, in the current study, we did not determine any significant alteration in the RNFL thickness values of the four quadrants and the foveal thickness in migraine patients without aura compared with the healthy controls. The mean disease duration was more than seven years in the migraine group, but we did not determine any structural alteration in the posterior ocular structures.

Although migraine is a periodic disease, its chronic nature might cause permanent structural abnormalities involving not only the brain but also the retina. These alterations have been associated with vascular alterations. However, the data regarding the OCTA results in patients with migraine is limited. Chang et al. [25] compared the VDs of the macula and optic nerve in migraine patients with and without aura and reported that the FAZ area was significantly larger in migraine patients with aura compared with the healthy controls, and the foveal VD was decreased in migraine patients with aura compared with the migraine patients without aura. The superior peripapillary VD of the optic nerve was also reduced in migraine patients with aura compared with those without aura. As mentioned above, Acer et al. [20] reported that the ocular pulse amplitude did not significantly differ between the migraine patients without aura and control cases. Very recently, Ulusoy et al. [26] determined that on macular OCTA, the superficial and deeper retinal foveal VDs were significantly lesser in the migraine patients with and without aura compared with the control cases. Moreover, the authors noted that the vascular alterations were more common with an enlarged FAZ size in migraine cases with aura, in which WMHs were detected. To the best of our knowledge, this is one of the first studies evaluating migraine patients without aura using OCTA. We determined a significant increase in the FAZ size, with a significant decrease in the superficial and deep VDs of the macula. The results are highly important to elucidate the pathophysiological mechanisms of this chronic, recurrent, common disease.

Another important finding of this study is that we determined a significant correlation between some clinical features of migraine patients and vascular alterations observed in this disease. Migraine is one of the leading causes of disability in the general population, and MIDAS scores have great importance in defining migraine-related disabilities [15, 27].

WMHs detected on MRI have been suggested as indicators of vascular abnormalities, especially in migraine cases with aura. However, we did not determine any clinical difference between the migraine patients with and without WMHs nor did we observe any alteration in the OCTA results in migraine patients without aura. Galli et al. [28] also reported that there was no clinical parameter, except age, that was in close relationship with such alterations in MRI in migraineur women. In this respect, the pathophysiological aspect and significance of WMHs seem to be not fully recognized yet and there is a need for novel studies.

In conclusion, in this study, we did not determine any alteration in the posterior ocular structures in migraine patients without aura. We also did not observe any changes in the optic disc VDs of these patients. However, there was a significant increase in the FAZ size and decrease in the macular vasculature in migraine patients without aura. We also determined that these alterations were associated with age and the MIDAS scores of the patients. Nevertheless, there was no significant difference in the clinical and ocular features of migraine patients with and without WMHs. Therefore, we suggest that for not only migraine with aura but also migraine without aura, neurovascular structures play an important role in pathogenesis, and novel studies are warranted to elucidate the alterations in these structures and determine the significance of WMHs in these patient groups.

The present study has several limitations. The first concerns the small sample size. Another limitation is that since this was an exploratory study, no adjustment was made for multiple comparisons; therefore, we were not able to draw definite conclusions. Considering that it is almost impossible to determine the exact role of vascular involvement and increased FAZ size in the pathogenesis of migraine using cross-sectional studies, we are currently performing a follow-up study to better explain our findings.

## Figures and Tables

**Table 1:** Demographic features of the study participants.

Parameters	Healthy controls (*n* = 43)	Migraine without WMHs (*n* = 37)	Migraine with WMHs (*n* = 29)	*P*
Age (years)	36.88 ± 8.23	38.43 ± 7.61	37.44 ± 7.82	0.463
Gender (F/M)	32/11	29/8	24/5	0.11

F: female, M: male, WMH: white matter hyperintensities.

**Table 2:** Comparison of the general characteristics of the migraine patients with and without WMHs.

	Migraine without WMHs (*n* = 37)	Migraine with WMHs (*n* = 29)	*P*
Disease duration (years)	7.05 ± 3.46	8.41 ± 3.39	0.29
Attack frequency/last month	8.05 ± 3.16	6.27 ± 3.64	0.11
Attack duration (hours)	48.45 ± 11.76	51.17 ± 10.03	0.57
VAS	6.91 ± 1.32	6.96 ± 1.52	0.89
MIDAS	16.19 ± 6.03	15.65 ± 6.22	0.78
VEP (latency)	109.79 ± 7.25	108.86 ± 11.19	0.56

WMHs: white matter hyperintensities; VAS: visual analogue scale; MIDAS: migraine disability assessment score; VEP: visual evoked potential.

**Table 3:** Comparison of the GCC count, foveal thickness, and RNFL thickness between the groups.

	Healthy controls (*n* = 43)	Migraine without WMHs (*n* = 37)	Migraine with WMHs (*n* = 29)	*P*
GCC superior (*μ*m)	110.34 ± 12.01	110.35 ± 14.45	108.36 ± 8.58	0.59
GCC inferior (*μ*m)	109.58 ± 10.55	110.25 ± 9.38	105.15 ± 8.35	0.72
Foveal thickness (*μ*m)	263.76 ± 21.29	263.85 ± 21.30	263.51 ± 21.16	0.96
RNFL (*μ*m)	114.47 ± 11.71	109.75 ± 9.24	111.01 ± 10.25	0.81
RNFL (upper) (*μ*m)	117.62 ± 10.81	113.04 ± 10.47	113.93 ± 10.38	0.76
RNFL (lower) (*μ*m)	107.32 ± 11.38	101.71 ± 10.11	104.01 ± 9.94	0.34

WMHs: white matter hyperintensities; GCC: ganglion cell complex; RNFL: retinal nerve fiber layer.

**Table 4:** Comparison of the optic disc VDs between the groups.

	Healthy controls (*n* = 43)	Migraine without WMHs (*n* = 37)	Migraine with WMHs (*n* = 29)	*P*
Optic disc superficial superior VD (mm^−1^)	54.48 ± 6.07	48.47 ± 5.13	47.17 ± 7.03	0.40
Optic disc superficial inferior VD (mm^−1^)	53.46 ± 5.83	50.83 ± 4.84	51.13 ± 6.49	0.84
Optic disc deep superior VD (mm^−1^)	54.51 ± 4.66	52.66 ± 6.96	51.96 ± 7.18	0.82
Optic disc deep inferior VD (mm^−1^)	56.84 ± 6.10	52.54 ± 5.91	52.31 ± 6.78	0.86

VD: vessel density.

**Table 5:** Comparison of the FAZ and superficial and deep macular VDs between the groups.

	Healthy controls (*n*: 43)	Migraine without WMHs (*n*: 37)	Migraine with WMHs (*n*: 29)	*P*
FAZ size (mm^2^)	0.24 ± 0.12	0.35 ± 0.11	0.36 ± 0.12	**0.034**
Superficial macular VD-whole area (mm^−1^)	47.22 ± 3.13	40.33 ± 4.32	39.82 ± 5.14	**0.001**
Superficial macular VD-inner layer (mm^−1^)	41.87 ± 4.64	35.64 ± 6.44	36.00 ± 6.21	**0.001**
Superficial macular VD-outer layer (mm^−1^)	53.05 ± 3.77	42.95 ± 4.97	41.50 ± 3.79	**0.001**
Deep macular VD-whole area (mm^−1^)	46.22 ± 4.61	36.62 ± 4.57	35.39 ± 4.78	**0.001**
Deep macular VD-inner layer (mm^−1^)	42.22 ± 3.13	33.10 ± 7.80	32.01 ± 5.57	**0.001**
Deep macular VD-outer layer (mm^−1^)	50.36 ± 6.95	38.56 ± 7.86	37.32 ± 4.69	**0.001**

FAZ: foveal avascular zone; VD: vessel density.

**Table 6:** Correlation analysis of the FAZ size with age, and VAS and MIDAS scores among the migraine patients.

	FAZ		Superficial macular VD		Deep macular VD	
	*r*	*P*	*R*	*P*	*r*	*P*
Age	**0.202**	**0.007**	−**0.162**	**0.032**	−**0.154**	**0.042**
Disease duration (years)	0.01	0.875	−0.067	0.441	−0.028	0.751
Attack frequency/last month	0.02	0.873	−0.036	0.771	−0.086	0.491
Attack duration (hours)	0.14	0.232	−0.051	0.68	−0.119	0.341
VAS	**0.279**	**0.023**	−**0.386**	**0.035**	−**0.254**	**0.018**
MIDAS	**0.275**	**0.014**	−**0.289**	**0.028**	−**0.331**	**0.041**
VEP (latency)	0.01	0.91	−0.018	0.841	−0.025	0.773

FAZ: foveal avascular zone; VD: vessel density; VAS: visual analogue scale; MIDAS: migraine disability assessment score; VEP: visual evoked potential.

## Data Availability

The data used to support the findings of this study are available from the corresponding author upon request.
